# No-Touch Automated Disinfection System for Decontamination of Surfaces in Hospitals

**DOI:** 10.3390/ijerph17145131

**Published:** 2020-07-16

**Authors:** Patryk Tarka, Aneta Nitsch-Osuch

**Affiliations:** Department of Social Medicine and Public Health, Medical University of Warsaw, Oczki 3, 02-007 Warsaw, Poland; anitsch@wum.edu.pl

**Keywords:** no-touch automated disinfection, hospital-acquired infections, peroxone, ATP bioluminescence assay, microbiological assay

## Abstract

Background: Hospital-acquired infections (HAIs) remain a common problem, which suggests that standard decontamination procedures are insufficient. Thus, new methods of decontamination are needed in hospitals. Methods: We assessed the effectiveness of a no-touch automated disinfection (NTD) system in the decontamination of 50 surfaces in 10 hospital rooms. Contamination of surfaces was assessed with a microbiological assay and an ATP bioluminescence assay. Unacceptable contamination was defined as > 100 colony forming units/100 cm^2^ in the microbiological assay, and as ≥ 250 relative light units in the ATP assay. Results: When measured with the microbiological assay, 11 of 50 surfaces had unacceptable contamination before NTD, and none of the surfaces had unacceptable contamination after NTD (*p* < 0.001). On the ATP bioluminescence assay, NTD decreased the number of surfaces with unacceptable contamination from 28 to 13, but this effect was non-significant (*p* = 0.176). On the microbiological assay taken before NTD, the greatest contamination exceeded the acceptable level by more than 11-fold (lamp holder, 1150 CFU/100 cm^2^). On the ATP bioluminescence assay taken before NTD, the greatest contamination exceeded the acceptable level by more than 43-fold (Ambu bag, 10,874 RLU). Conclusion: NTD effectively reduced microbiological contamination in all hospital rooms. However, when measured with the ATP bioluminescence assay, the reduction of contamination was not significant.

## 1. Introduction

Hospital-acquired infections (HAIs) are a serious healthcare problem, with about 2 million cases diagnosed every year both in the USA and the European Union [1,2,3]. In the USA alone, about $28–45 billion are spent every year to treat patients with HAIs [4].

Pathogens causing HAIs can survive for weeks or months on non-disinfected surfaces, such as bed rails, medical equipment, phones, keyboards, and patients’ files [5,6,7,8,9]. These pathogens are often multidrug-resistant, including methicillin-resistant *S. aureus*, carbapenem-resistant *P. aeruginosa*, vancomycin-resistant *E. faecium,* and extended-spectrum β-lactamase-resistant *K. pneumoniae* [10,11]. It seems that using standard cleaning products and complying with prevention practices, such as proper hand hygiene, are not sufficient to stop HAIs [12]. Therefore, additional methods of decontamination are needed in hospitals.

**Figure 1:**
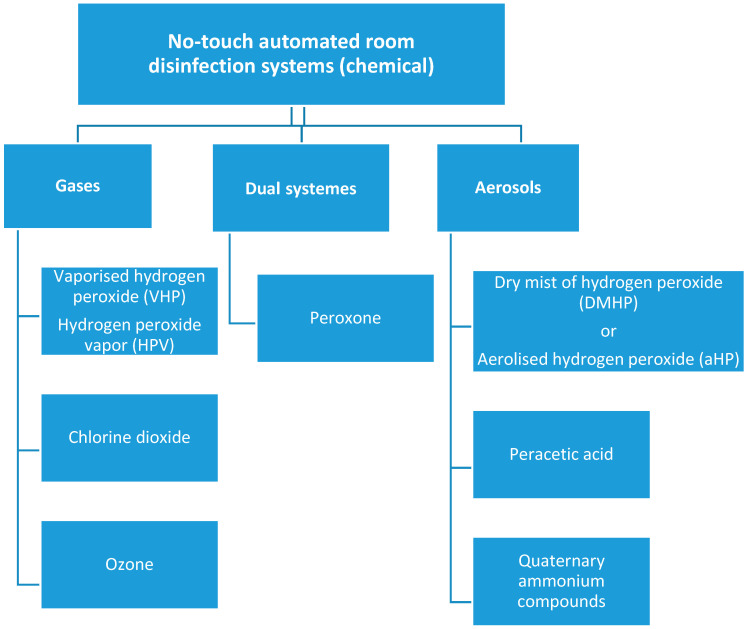
Chemical no-touch room disinfection systems.

No-touch automated disinfection (NTD) is a promising approach to reduce HAIs. NTD systems are based on vaporised hydrogen peroxide (VHP), hydrogen peroxide vapor (HPV), chlorine dioxide, gaseous ozone, dry mist of hydrogen peroxide (DMHP), or aerosolised hydrogen peroxide (aHP), often complemented with silver cations, aerosolised peracetic acid, quaternary ammonium compounds, high-intensity narrow-spectrum (405 nm) light, and pulsed-xenon UV (PX-UV) radiation [13,14,15]. Among the currently available systems, ultraviolet light devices PX-UV and ultraviolet light-C allow to achieve a log10 reduction in pathogen content of 2–4, aHP systems of ≤ 4, whereas a log10 reduction greater than six is reported for vapor chlorine dioxide, hydrogen peroxide-based VHP, HPV, and dual peroxone systems [16,17,18]. Chemical NTDs are presented in Figure 1. From all NTD systems, technologies based on advanced oxidation processes are of particular interest. Hydrogen peroxide is a potent oxidant, demonstrating stronger biocidal activity in the form of vapor than in aerosol [15]. In advanced oxidation technologies, hydrogen peroxide is combined with ozone to create peroxone—a highly potent oxidative compound [19].

Data on the performance of peroxone-based NTD systems in hospital settings are limited. Therefore, we assessed the effectiveness of an NTD system in decontamination of critical surfaces in different hospital rooms using two methods: classical microbiological assay—demanding time to obtain results of bacteria growth—and fast ATP bioluminescence assay.

## 2. Material and Methods

### 2.1. Study Design and Sample Collection

This study was conducted in an oncological hospital in Poland. Contamination of five different surfaces in 10 hospital rooms (see Table 1) was measured before and after NTD with an ATP bioluminescence assay and a microbiological assay. The samples for both assays were collected on different days in September 2019.

According to the manufacturer’s instructions, before NTD, all hospital rooms were cleaned and disinfected with two types of cleaning solution: 55% ethanol + quaternary ammonium propionate—for small surfaces (holders, tables, medical equipment, etc.), and N-(3-aminopropyl)-N-dodécylopropane-1,3-diamine + dodecyl dimethyl ammonium chloride—for large surfaces (floors). The samples were collected up to 15 min after the initial cleaning.

For NTD, we used the system which sprays a peroxone vapor, a combination of hydrogen peroxide and ozone, to decontaminate all surfaces.

The duration of decontamination was one hour each time. The decontamination cycle consisted of the following phases: hydrogen peroxide phase, ozone phase—in which peroxone was formed, and one-hour contact phase. Next, the rooms were aerated for another hour.

Technical properties of NTD system used in the study were as follows:

- Disinfectant compound usage: 8 mL/m^3^
- Particle size generated by the device: 5–8 µm (dry mist)
- Hydrogen peroxide concentration: 7.5%
- Maximum ozone concentration: 3 ppm
- Contact time—1 h.
- Aerating time—1 h.

NTD system user guidelines strictly prohibit human attendance during the procedure. In our study, according to the guidelines, during NTD procedure the rooms were empty. The staff was allowed to enter the rooms after 2 h, when hydrogen peroxide concentration reached levels below 1 ppm. The samples were taken immediately after the aeration (i.e., 2 h after peroxone was formed).

### 2.2. ATP Bioluminescence Assays

ATP bioluminescence assays show general organic contamination such as bacteria, food, or human secretions and excretions [20]. These assays are based on the chemiluminescence properties of a luciferin-luciferase reagent, which emits light in the presence of ATP [21]. We used 3M Clean-Trace ATP Surface Test swabs (3M Company, St. Paul, MN, USA) to sample surfaces, according to the manufacturer’s recommendations. All surfaces were swabbed for 30 s, and the swabs were returned to the swab device. The ATP tests were read immediately or within 1 h of collection. The samples were analysed with a 3M Clean-Trace NGi Luminometer (3M Company, St. Paul, MN, USA), and the results were reported in relative light units (RLU). In a hospital setting, RLU > 250 is considered as unacceptable contamination [22].

### 2.3. Microbiological Assay

Due to the fact that most of the surfaces assessed in our study were not flat, we decided to use 3M™ Petrifilm™ plates (London, ON, Canada) [23]. Each plate (contact surface 20 cm^2^) contained a build-in grid to facilitate colony counting, water-soluble gelling agent, nutrients, and red dye indicator. The detailed composition of the medium was not provided by the manufacturer. In order to neutralise the disinfectants used during initial cleaning phase, each plate was hydrated with 1 cm^3^ of sterile disinfectant neutralizer: broth 1000 cm^3^, Tween 80 30.0 g, soy lecithin 3.0 g, L-histidine 1.0 g, thiosulfate 0.5 g. After the hydration, the plates were left for 1 h in room temperature and the gel was formed.

During the sample collection, the plates were pressed on each surface (500 g/cm^2^) for 10 s, with no side movements. The plates were incubated at 37 °C for 48 h under aerobic conditions. Then, the growth of micro-organisms was measured in colony-forming units (CFU) per 100 cm^2^ [24]. Values >100 CFU/100 cm^2^ were regarded as unacceptable contamination.

### 2.4. Statistical Analysis

Values from the two assays were presented for all surfaces before and after NTD. We used the following cut-offs for unaccepted contamination: ≥250 RLU in the ATP assay, and >100 CFU/100 cm^2^ in the microbiological assay. The McNemar test with continuity correction was used to assess whether NTD decreased the number of surfaces with unacceptable contamination significantly. The Wilcoxon test was used to compare the differences in median contamination before and after NTD. *p* < 0.05 was considered significant. R software (version 3.52, R Foundation for Statistical Computing, Vienna, Austria) was used for all calculations.

## 3. Results

**Figure 2:**
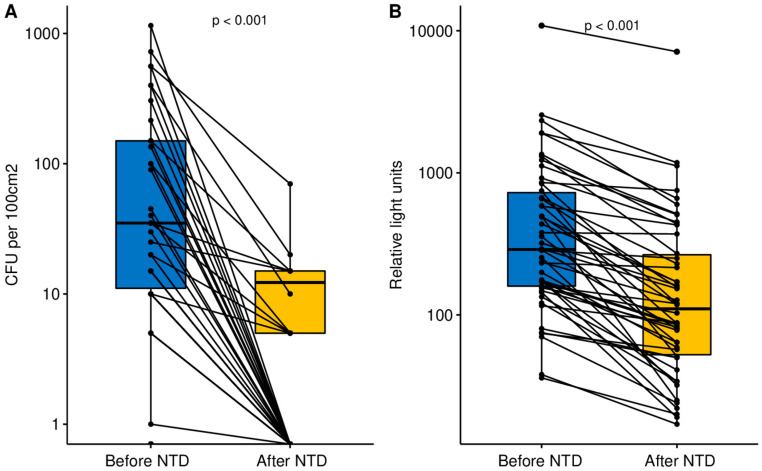
Contamination before and after no-touch disinfection (NTD) in the microbiological (**A**) and ATP bioluminescence (**B**) assays. Middle bars show median; upper and lower bars show interquartile range.

In total, 50 surfaces from 10 rooms were tested. The results are presented in Figure 2 and Table 1, in which unacceptable contamination is shown in bold.

### 3.1. Microbiological Assay

Before NTD, unacceptable contamination was detected on 11 of 50 surfaces. Contamination was acceptable on all tested surfaces in the Patient Recovery Room 2, Operating Room 1, and Ward no. 116. The greatest contamination (1150 CFU/100 cm^2^) was detected on a treatment lamp holder. After NTD, contamination was acceptable on all surfaces in all rooms. The greatest contamination (70 CFU/100 cm^2^) was detected on the phone in the patient recovery room. NTD decreased significantly the number of surfaces with unacceptable contamination measured with the microbiological assay: from 11/50 to 0/50 (*p* < 0.001). Similarly, the reduction in median contamination on all surfaces was also significant (*p* < 0.001, Figure 2A)

### 3.2. ATP Bioluminescence Assay

Before NTD, unacceptable contamination was detected on 28 of 50 surfaces. None of the rooms had acceptable contamination on all surfaces. The greatest contamination was detected on the phone in Operating Room 4 (2331 RLU), the phone in the Patient Recovery Room 1 (2552 RLU), and the Ambu bag in the Patient Recovery Room 1 (10,874 RLU). After NTD, acceptable contamination on all surfaces was detected in the Endoscopy Unit, Ward no. 116, and in the Patient Recovery Room 2. The greatest contamination was detected on the phone (1178 RLU), bed railing (1119 RLU), and Ambu bag in the Patient Recovery Room 1 (7123 RLU). NTD decreased, non-significantly, the frequency of surfaces with unacceptable contamination measured with the ATP bioluminescence assay: from 28/50 to 13/50 (*p* = 0.176). However, the reduction in median contamination on all surfaces was significant (*p* < 0.001, Figure 2B).

## 4. Discussion

The NTD system used in this study reduced microbiological contamination in different hospital rooms. NTD decreased significantly the number of surfaces with unacceptable contamination measured with the microbiological assay, but not with the ATP bioluminescence assay.

The efficiency of decontamination depended on the given surface. On some surfaces, contamination measured with the ATP assay decreased only slightly after NTD—for example, on an operating lamp holder (379 vs. 371 RLU) or X-ray apparatus keyboard (854 vs. 751 RLU). On other surfaces, such as a remote control, contamination decreased considerably (495 vs. 25 RLU). Such differences may be associated with the type of material of the decontaminated surface. In the study by Eschlbeck et al. [25], it was observed that the rate of decontamination with hydrogen peroxide depended on the hydrophobicity of the material, while its porosity played a minor role. Also, the amount of the agent used in NTD may influence the effectiveness of the process, as shown in the study by Horn and Niemeyer [26], in which better outcomes were achieved by increasing the volume of peracetic acid.

In our study, the number of surfaces with unacceptable microbiological contamination before NTD was much higher when assessed with the ATP bioluminescence assay than the microbiological assay (28 vs. 11 surfaces). The greatest contamination measured with the microbiological assay exceeded the acceptable level by more than 11-fold (treatment lamp holder, 1150 CFU/100 cm^2^), and, for the ATP bioluminescence assay, by more than 43-fold (Ambu bag, 10,874 RLU). After decontamination with the NTD system, the number of surfaces with unacceptable contamination was still much higher when measured with the ATP bioluminescence assay than with the microbiological assay (13 vs. 0).

Several studies have compared ATP bioluminescence and microbiological assays. In a study evaluating over 80 different surfaces, a microbiological assay showed unacceptable contamination on about 20% of surfaces before cleaning, and on about 6% after cleaning; the respective figures for an ATP assay were considerably higher: 50% and 20%, respectively [27]. In another study assessing the effects of decontamination with vaporised hydrogen peroxide, a standard microbiological assay, but not an ATP bioluminescence assay, showed multiple log-reductions in contamination [28]. Those investigators concluded that the ATP bioluminescence assay should not be an alternative to standard microbiological methods [28]. Our study is in line with these findings. Indeed, in a review by Nante et al. [29], from 14 papers assessing correlation between RLU and aerobic colony counts (ACC), three of the studies showed no correlation, and four of them showed only poor/moderate correlation. However, because different ATP assays with no standardised threshold are available, comparisons between studies are challenging. Of note, a new standard EN 17272:2020 was approved by the Technical Committee 216 of European Committee for Standardisation (CEN/TC 216), describing the methods to determine the disinfectant activity of automated processes for distributing chemicals by air diffusion [30]. The unified standard will provide guidance for future research, making future studies more comparable.

ATP assays are not optimal for estimating of the risk of infection. These assays cannot distinguish between pathogenic and non-pathogenic bacteria and between live and dead cells. The relationship between the level of residual surface contamination and the risk of infection after conventional disinfection has not been extensively studied. However, multiple studies revealed that subsequent patients occupying the hospital room after infected individuals are at increased risk of infection [31,32,33,34,35]. Probably the conventional methods do not sufficiently prevent from disease transmission. Bshabshe et al. [36] showed that NTD can reduce that risk. In another study, Anesi et al. [37] observed a reduction in *Clostridioides (Clostridium) difficile* rate from 1.73 to 0.93 cases per 1000 patient-days in the 12 months after NTD implementation. Being especially valuable in heavy patient overload, when the risk of disease transmission is high, NTD systems have been recommended in SARS-CoV-2 pandemic management [38].

In assessing the effectiveness of cleaning and decontamination methods, particular consideration should be paid to potential human errors, e.g., improper formulation or contact time of the agent, sensitive area omission, etc. [39]. NTD systems eliminate the impact of the human factor. Our study is in line with other trials [40] confirming that NTD can effectively inactivate pathogenic microorganisms while conventional methods remain insufficient.

The limitations of our study need to be mentioned. First, the sample size, i.e., the number of surfaces and hospital rooms that were evaluated was small. Second, the chemical compounds used in our study before NTD can interfere with ATP-based assays [41]. Also, we did not identify the species present in the examined rooms before and after using NTD system. Qualitative methods of assessment would be particularly valuable in assessing the risk of HAI before and after decontamination, however, quantitative methods seem to support the effectiveness of NTD system as it reduced the contamination levels below standards commonly used in clinical settings.

Finally, in microbiological tests, we used the value >100 CFU/100 cm^2^ as a threshold of unacceptable contamination, which is not common in other research. Some authors suggest for clinical surface hygiene assessments the same threshold as for the food industry (>250 CFU/100 cm^2^) [42]. This standard has been implemented in many studies [43,44,45,46]; however, it is not clear if it is adequate for the prevention of disease transmission in healthcare facilities [47]. Hence, we decided to use a stricter standard [24]. According to Carling et al. [48], only surfaces with undetectable levels of biological contamination (0 CFU) achieved after documented cleaning can be considered successfully decontaminated.

## 5. Conclusions

In summary, our study showed that NTD is a feasible method of decontamination: when added to standard decontamination procedures, it can further decrease the risk of HAIs. It seems that ATP bioluminescence assays with standard cut-off are not valid for assessing the effectiveness of NTD. Further studies are needed to standardise the cut-off values.

## Figures and Tables

**Table 1:** Surface contamination before and after no-touch disinfection in different hospital rooms.

		Microbiological Assay (CFU/100 cm^2^)		ATP Bioluminescence Assay (RLU)	
Room	Surface	Before NTD	After NTD	Before NTD	After NTD
**Operating Room 4**	Operating lamp holder	15	0	**379**	**371**
	Upper surface of operating table	5	0	172	85
	Anaesthetic machine (table)	45	0	159	78
	Mayo table	0	0	172	64
	Phone	**560**	0	**2331**	**599**
**Treatment Room in Ward I**	Treatment lamp holder	**1150**	0	**1119**	**508**
	Upper surface of mattress	10	5	155	103
	Treatment table	**150**	0	**840**	120
	Sink	15	0	176	22
	Locker holder under sink	5	0	**587**	87
**Patient Recovery Room 1**	Phone	**560**	70	**2552**	**1178**
	Bed remote control	10	0	**668**	229
	Bed railing	**215**	0	**1905**	**1119**
	Ambu bag	**400**	10	**10,874**	**7123**
	Sink	10	0	**253**	32
**Endoscopy Unit: automatic disinfector room**	Sink	0	0	70	24
	Locker holder	**400**	0	**356**	64
	Remote control of disinfector	0	0	**746**	51
	Inside of automatic disinfector	0	0	144	19
	Tap above sink	10	0	**320**	161
**Endoscopy Unit: examination room**	Video track endoscopy keyboard	**305**	0	**656**	156
	Blood pressure cuff	40	0	**1308**	**270**
	Anaesthesiological table	90	0	74	57
	Storage	35	5	**436**	171
	Anaesthesiological table handle	**725**	20	**488**	170
**Operating Room 1**	Upper surface of operating table	0	0	175	87
	Operating lamp holder	0	0	75	50
	Computer mouse	25	15	**1230**	**514**
	X-ray apparatus keyboard	35	15	**854**	**751**
	Ambu bag	0	0	116	88
**Operating Room 2**	Infusion pump remote control panel	0	0	**918**	**450**
	Ambu bag	**135**	0	**1349**	**437**
	Anaesthesiological table	0	0	148	82
	Blood pressure cuff	20	0	121	34
	Operating table remote control panel	0	0	**583**	**430**
**Ward no. 116**	Calling staff pilot	1	0	80	50
	Mattress	20	5	**291**	153
	Toilet flush button	0	0	38	17
	Bedside table	100	5	235	127
	Bed railing	30	0	232	215
**Patient Recovery Room 2**	Infusion pump control panel	0	0	160	120
	Sink	0	0	**286**	80
	Bedside table	0	0	**495**	118
	Cardiomonitor screen	0	0	**284**	250
	Handle locker	0	0	173	34
**Treatment Room Ward I**	Bed remote control	5	0	**495**	25
	Sink	10	0	36	20
	ECG apparatus keyboard	**150**	15	**1905**	**661**
	Treatment lamp holder	15	0	134	41
	Upper surface of mattress	100	0	199	59

Notes: unacceptable contamination is shown in bold.

## References

[1] Klevens R.M., Edwards J.R., Richards C.L. (2007). Estimating healthcare-associated infections and deaths in U.S. hospitals, 2002. Public Health Rep..

[2] Anderson R.N. (2001). Deaths: Leading causes for 1999. Natl. Vital. Stat. Rep..

[3] Cassini A., Plachouras D., Eckmanns T., Sin M.A., Blank H.-P., Ducomble T., Haller S., Harder T., Klingeberg A., Sixtensson M. (2016). Burden of Six Healthcare-Associated Infections on European Population Health: Estimating Incidence-Based Disability-Adjusted Life Years through a Population Prevalence-Based Modelling Study. PLoS Med..

[4] Scott R., Polock D.A., Stone P.W. (2009). The Direct Medical Costs of Healthcare-Associated Infections in US Hospitals and the Benefits of Prevention. Division of Healthcare Quality Promotion, National Center for Preparedness, Detection, and Control of Infectious Diseases, Coordinating Center for Infectious Diseases, Centers for Disease Control and Prevention.

[5] Kramer A., Schwebke I., Kampf G. (2006). How long do nosocomial pathogens persist on inanimate surfaces? A systematic review. BMC Infect. Dis..

[6] Sole M.L., Poalillo F.E., Byers J.F., Ludy J.E. (2002). Bacterial growth in secretions and on suctioning equipment of orally intubated patients: A pilot study. Am. J. Crit. Care.

[7] Catalano M., Quelle L.S., Jeric P.E., Di Martino A., Maimone S.M. (1999). Survival of Acinetobacter baumannii on bed rails during an outbreak and during sporadic cases. J. Hosp. Infect..

[8] Heyba M., Ismaiel M., Alotaibi A., Mahmoud M., Baquer H., Safar A., Al-Sweih N., Al-Tailr A. (2015). Microbiological contamination of mobile phones of clinicians in intensive care units and neonatal care units in public hospitals in Kuwait. BMC Infect. Dis..

[9] Hartmann B., Benson M., Junger A., Quinzio L., Rohring R., Fengler B., Farber U.W., Wille B., Hempelmann G. (2004). Computer keyboard and mouse as a reservoir of pathogens in an intensive care unit. J. Clin. Monit. Comput..

[10] Sievert D.M., Ricks P., Edwards J.R., Schneider A., Patel J., Srinivasan A., Kallen A., Limbago B., Fridkin S.F. (2013). National Healthcare Safety Network (NHSN) Team and Participating NHSN Facilities. Antimicrobial-resistant pathogens associated with healthcare-associated infections: Summary of data reported to the National Healthcare Safety Network at the Centers for Disease Control and Prevention, 2009–2010. Infect. Control Hosp. Epidemiol..

[11] Hidron A.I., Edwards J.R., Patel J., Horan T.C., Sievert D.M., Pollock D.A., Fridkin S.F. (2008). National Healthcare Safety Network Team; Participating National Healthcare Safety Network Facilities. Infections: Annual summary of data reported to the National Healthcare Safety Network at the Centers for Disease Control and Prevention, 2006–2007. Infect. Control Hosp. Epidemiol..

[12] Wilson A.P.R., Smyth D., Moore G., Singleton J., Jackson R., Gant V., Jeanes A., Shaw S., James E., Cooper B. (2011). The impact of enhanced cleaning within the intensive care unit on contamination of the near-patient environment with hospital pathogens: A randomized crossover study in critical care units in two hospitals. Crit. Care Med..

[13] Dancer S.J. (2014). Controlling hospital-acquired infection: Focus on the role of the environment and new technologies for decontamination. Clin. Microbiol. Rev..

[14] Han J.H., Sullivan N., Leas B.F., Pegues D.A., Kaczmarek J.L., Umscheid C.A. (2015). Cleaning Hospital Room Surfaces to Prevent Health Care-Associated Infections: A Technical Brief. Ann. Intern. Med..

[15] Otter J.A., Yezli S., Perl T.M., Barbut F., French G.L. (2014). 17—A guide to no-touch automated room disinfection (NTD) systems. Decontamination in Hospitals and Healthcare.

[16] Casini B., Tuvo B., Cristina M.L., Spagnolo A.M., Totaro M., Baggiani A., Privitera G.P. (2019). Evaluation of an ultraviolet C (UVC) light-emitting device for disinfection of high touch surfaces in hospital critical areas. Int. J. Environ. Res. Public Health.

[17] Elgujja A., Altalhi H., Ezreqat S. (2020). Review of the efficacy of ultraviolet C for surface decontamination. J. Nat. Sci. Med..

[18] Otter J.A., Yezli S., Perl T.M., Barbut F., French G.L. (2013). The role of “no-touch” automated room disinfection systems in infection prevention and control. J. Hosp. Infect..

[19] Xu X., Goddard W.A. (2002). Peroxone chemistry: Formation of H2O3 and ring-(HO2) (HO3) from O3/H2O2. Proc. Natl. Acad. Sci. USA.

[20] Moore G., Smyth D., Singleton J., Wilson P. (2010). The use of adenosine triphosphate bioluminescence to assess the efficacy of a modified cleaning program implemented within an intensive care setting. Am. J. Infect. Control.

[21] Shama G., Malik D. (2013). The uses and abuses of rapid bioluminescence-based ATP assays. Int. J. Hydrogen Environ. Health.

[22] European Committee for Standardization (1993). CEN/TC 243—Cleanroom Technology. https://standards.iteh.ai/catalog/tc/cen/9f58539c-5394-4f44-afe3-8528a2e35e33/cen-tc-243.

[23] Rawlinson S., Ciric L., Cloutman-Green E. (2019). How to carry out microbiological sampling of healthcare environment surfaces? A review of current evidence. J. Hosp. Inf..

[24] Draft European Standard CEN/TC/243/WG2/1993. https://www.hex-group.eu/en_BE/biocontamination-control/.

[25] Eschlbeck E., Seeburger C., Kulozik U. (2020). Spore inactivation on solid surfaces by vaporized hydrogen peroxide—Influence of carrier material surface properties. J. Food. Sci..

[26] Horn H., Niemeyer B. (2020). Aerosol disinfection of bacterial spores by peracetic acid on antibacterial surfaces and other technical materials. Am. J. Infect. Control.

[27] Huang Y.S., Chen Y.C., Chen M.L., Cheng A., Hung I.C., Wang J.T., Sheng W.H., Chang S.C. (2015). Comparing visual inspection, aerobic colony counts, and adenosine triphosphate bioluminescence assay for evaluating surface cleanliness at a medical center. Am. J. Infect. Control..

[28] Colbert M.E., Gibbs S.G., Schmid K.K., High R., Lowe J.J., Chaika O., Smith P.W. (2015). Evaluation of adenosine triphosphate (ATP) bioluminescence assay to confirm surface disinfection of biological indicators with vaporised hydrogen peroxide (VHP). Healthc. Infect..

[29] Nante N., Ceriale E., Messina G., Lenzi D., Manzi P. (2017). Effectiveness of ATP bioluminescence to assess hospital cleaning: A review. J. Prev. Med. Hyg..

[30] EN 17272:2020 Chemical Disinfectants and Antiseptics—Methods of Airborne Room Disinfection by Automated Process —Determination of Bactericidal, Mycobactericidal, Sporicidal, Fungicidal, Yeasticidal, Virucidal and Phagocidal Activities. https://standards.cen.eu/dyn/www/f?p=204:110:0::::FSP_LANG_ID,FSP_PROJECT:25,62318&cs=10D54BE215B9679AD9914C42C71BA77CE.

[31] Weber D.J., Rutala W.A., Miller M.B., Huslage K., Sickbert-Bennett E. (2010). Role of hospital surfaces in the transmission of emerging health care-associated pathogens: Norovirus, Clostridium difficile, and Acinetobacter species. Am. J. Infect. Control.

[32] Datta R., Platt R., Yokoe D.S., Huang S.S. (2011). Environmental cleaning intervention and risk of acquiring multidrug-resistant organisms from prior room occupants. Arch. Intern. Med..

[33] Huang S.S., Datta R., Platt R. (2006). Risk of acquiring antibiotic-resistant bacteria from prior room occupants. Arch. Intern. Med..

[34] Drees M., Snydman D., Schmid C. (2008). Prior environmental contamination increases the risk of acquisition of vancomycin-resistant enterococci. Clin. Infect. Dis..

[35] Nseir S., Blazejewski C., Lubret R., Wallet F., Courcol R., Durocher A. (2011). Risk of acquiring multidrug-resistant Gram-negative bacilli from prior room occupants in the ICU. Clin. Microbiol. Infect..

[36] Bshabshe A.A., Joseph M.R.P., Assiri A., Omer H.A., Hamid M.E. (2020). A multimodality approach to decreasing ICU infections by hydrogen peroxide, silver cations, and compartmentalization. J. Infect. Public Health.

[37] Anesi A., Rognoni V., Accetta R., Ferrari M. Impact of hydrogen peroxide and silver cations dry-mist disinfection on the reduction of hospital acquired Clostridium difficile infections. Proceedings of the 28th European Congress of Clinical Microbiology and Infectious Diseases.

[38] Fudan Zhongshan Guidance of COVID-19 Prevention and Control. https://www.tencent.com/en-us/responsibility/combat-covid-19-hospital-guidance.html.

[39] Carling P.C., Von Beheren S., Kim P., Woods C. (2008). Healthcare Environmental Hygiene Study Group. Intensive care unit environmental cleaning: An evaluation in sixteen hospitals using a novel assessment tool. J. Hosp. Infect..

[40] Chiguer M., Maleb A., Amrani R., Abda N., Alami Z. (2019). Assessment of surface cleaning and disinfection in neonatal intensive care unit. Heliyon.

[41] Brown E., Eder A.R., Thompson K.M. (2010). Do surface and cleaning chemistries interfere with ATP measurement systems for monitoring patient room hygiene?. J. Hosp. Infect..

[42] Dancer S.J. (2004). How do we assess hospital cleaning? A proposal for microbiological standards for surface hygiene in hospitals. J. Hosp. Infect..

[43] Lewis T., Griffith C., Gallo M., Weinbren M. (2008). A modified ATP benchmark for evaluating the cleaning of some hospital environmental surfaces. J. Hosp. Infect..

[44] Mulvey D., Redding P., Robertson C., Woodall C., Kingsmore P., Bedwell D., Dancer S.J. (2011). Finding a benchmark for monitoring hospital cleanliness. J. Hosp. Infect..

[45] Smith P.W., Sayles H., Hewlett A., Cavalieri R.J., Gibbs S.G., Rupp M.E. (2013). A study of three methods for assessment of hospital environmental cleaning. Healthc. Infect..

[46] Ho Y.H., Wang L.S., Jiang H.L., Chang C.H., Hsieh C.J., Chang D.C., Tu H.Y., Chiu T.Y., Chao H.J., Tseng C.C. (2016). Use of a Sampling Area-Adjusted Adenosine Triphosphate Bioluminescence Assay Based on Digital Image Quantification to Assess the Cleanliness of Hospital Surfaces. Int. J. Environ. Res. Public. Health..

[47] Griffith C.J., Obee P., Cooper R.A., Burton N.F., Lewis M. (2007). The effectiveness of existing and modified cleaning regimens in a Welsh hospital. J. Hosp. Infect..

[48] Carling P.C., Perkins J., Ferguson J., Thomasser A. (2014). Evaluating a New Paradigm for Comparing Surface Disinfection in Clinical Practice. Infect. Control. Hosp. Epidemiol..

